# ATP-responsive Mn(ii)-based *T*_1_ contrast agent for MRI†

**DOI:** 10.1039/d3cc03430e

**Published:** 2023-09-19

**Authors:** Sellamuthu Anbu, Lawerence Kenning, Graeme J. Stasiuk

**Affiliations:** a Departments of Chemistry and Biomedical Sciences, University of Hull Cottingham Road Hull HU6 7RX UK bioinorg_anbu@yahoo.com; b School of Chemistry, University of East Anglia, Norwich Research Park Norwich NR4 7TJ UK; c MRI Centre, Royal Infirmary Hospital NHS Trust Anlaby Road Hull HU3 2JZ UK; d Department of Imaging Chemistry and Biology, School of Biomedical Engineering and Imaging Sciences, King's College London Fourth Floor Lambeth Wing, St Thomas’ Hospital London SE1 7EH UK graeme.stasiuk@kcl.ac.uk

## Abstract

A novel diacetylpyridylcarbohydrazide–DAPyCOHz-based manganese(ii) chelate with dipicolylamine/zinc(ii) (DPA/Zn^2+^) arms (MnL^DPA-Zn^2) was developed for adenosine triphosphate (ATP) responsive magnetic resonance imaging (MRI) *T*_1_ contrast applications. Compound 2 shows enhanced relaxivity (*r*_1_ = 11.52 mM^−1^ s^−1^) upon selective ATP binding over other phosphates.

## Abstract 2

A manganese(ii)-based ATP-responsive chelate was developed for MRI (*T*_1_) contrast applications.

## Graphical abstract

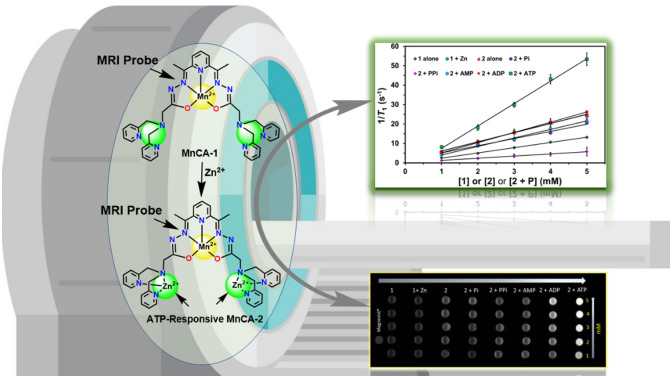

MRI is a unique and non-invasive technique for distinguishing between healthy and diseased tissues throughout the body, eliminating the need for invasive biopsies. While it often relies on Gd^3+^-based contrast agents (GdCAs) to generate high-resolution 3D images of cellular functions at a molecular level,^1*b*^ concern has arisen in recent years about using linear GdCAs for patients with renal impairment. These agents have been found to release toxic Gd^3+^ ions, which can cause serious adverse effects, including nephrogenic systemic fibrosis (NSF),^2^ a severe disorder characterised by scleroderma-like skin abrasions that occur in patients with kidney diseases.^3^ In response, researchers have turned their attention to developing safe alternatives to GdCAs, such as Mn^2+^-based contrast agents (MnCAs).^4^

Mn^2+^ is a biogenic metal ion that plays critical roles in several physiological and cellular (mitochondrial) functions, and its five unpaired electrons in the 3d subshell make it a promising candidate for enhancing MR images.^5^ Mn^2+^ involves maintaining normal ATP levels in the liver, heart, and brain mitochondria.^6^ Despite its superior biocompatibility and lower nephrotoxicity compared to GdCAs, no clinically available MnCA exists.^7^ Hence, research is required to develop novel MnCAs that exhibit improved chelating stability to serve as next-generation MRI contrast probes. One promising investigation avenue involves using SMART or responsive (MRI) contrast agents that maximise relaxivity by optimising electron spin properties, water accessibility, and time scales for molecular motion through the receptor-induced magnetisation effect (RIME).^8^ To date, only a few responsive MnCAs have been developed in the past two decades, all of which are redox-active/pH or Zn-sensitive^9–11^ but not anion or nucleotide-responsive MnCAs.

ATP, an essential anionic nucleotide, acts as a ubiquitous molecular reservoir of energy in living organisms and governs a wide array of cellular processes, including cell proliferation.^12^ In a noteworthy observation, many research groups have found that the external ATP concentration (0.1–0.7 mM) in cancerous cells can be up to 10^3^–10^4^ times greater than in healthy cells from the same tissue type.^13,14*a*^ Notably, extracellular ATP increases intracellular ATP levels, promoting rapid cancer cell migration, metastasis, and resistance to tyrosine kinase inhibitor-type anticancer drugs.^14^ Therefore, using responsive CAs to measure ATP levels in cancer cells represents a promising avenue to gain crucial insights into the phosphate-mechanism-accompanied cancer staging and progression and identify appropriate treatment options. To date, only two ATP-responsive GdCAs have been reported, but they suffer from a lack of ATP specificity over other nucleotides or inorganic pyrophosphate ions (PPi).^15^

Recently, we have explored the *in vivo* MRI contrasting potential of DAPyCOHz-based MnCA such as MnL^Me^ (Scheme 1),^1*a*^ which has a higher *T*_1_ relaxivity (*r*_1_ = 4.90 mM^−1^ s^−1^) than clinical GdCA such as Magnevist® and clears faster through the kidney and hepatobiliary system. This was attributed to the rigidity of the DAPyCOHz ligand (L^Me^) with carbohydrazide moiety that led to a kinetically stable bishydrated Mn^2+^ complex (*q* = 1.7).^1*a*^ In this work, for the first time, we have developed a novel Zn/ATP-responsive MnCA (MnL^DPA^, 1) by interlinking the Zn selective dipicolylamine (DPA) groups and kinetically stable Mn-DAPyCOHz chelate, in which former can be an ATP sensor/binder, and latter serves as MRI probe.

**Scheme 1:**
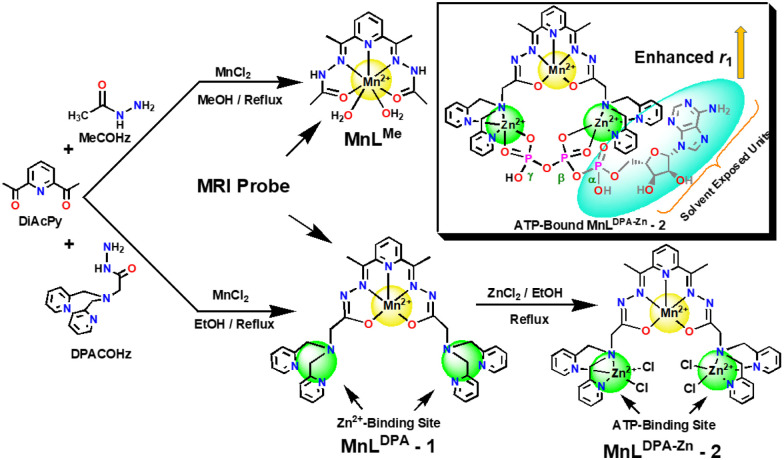
Synthesis of Zn^2+^/ATP-responsive MnCAs (1 and 2).

The Zn^2+^-responsive MnCA 1 was prepared by refluxing the dipicolylamine carbohydrazide DPACOHz with 2,6-diacetylpyridine and MnCl_2_ (2 : 1 : 1 molar ratio, respectively) in methanol using the innovative single-pot template (Schiff base) reaction strategy.^1*a*^ By using 1 as a precursor compound, we have achieved the proposed Zn^2+^ bound Mn^2+^-chelate (MnL^DPA-Zn^, 2) by stirring it with ZnCl_2_ (1 : 2 molar ratio) in ethanol at 323 K. All the synthesised compounds were characterised by elemental analysis and spectroscopic methods (Fig. S1–S4, ESI†). The UV-visible spectrum of the Mn^2+^-chelate with DPA arms 1 shows three absorption bands at 215, 257, and 274 nm in 50 mM HEPES buffer at pH = 7.3. These are attributed to the π–π* and n–π* transitions between the pyridyl moieties and electron-localised CONH functionalities in L^DPA^ (Fig. S5, ESI†). The HR-ESMS analyses (Fig. S3 and S4, ESI†) of 1 and 2 confirm the formation of mononuclear (Mn(ii)) and hetero-trinuclear (Mn(ii)–Zn_2_) complexes, where the spectral profiles display peaks at *m*/*z* of 723.2599 & 745.2404 and 959.0108, 924.0421 & 462.0210 corresponding to the expected [MnL^DPA^ + H^+^]^+^ & [MnL^DPA^ + Na^+^]^+^ and [MnL^DPA-Zn^-Cl^−^]^+^, [MnL^DPA-Zn^-(2Cl^−^) + H^+^]^+^ & [MnL^DPA-Zn^-(2Cl^−^)]^2+^ species, respectively. The resulting data confirms the proposed structures of Mn^2+^-chelates 1 and 2.

To establish the longitudinal proton relaxivity (*r*_1_) of 1 and 2, the *T*_1_ (longitudinal relaxation time) values at different concentrations of 1 and 2 have been measured by the inversion-recovery method (180° − *τ* − 90°) in 400 MHz (9.4 T) at 298 K. The *r*_1_ values of 1 and 2 were determined as 3.57 ± 0.2 and 5.27 ± 0.2 mM^−1^ s^−1^, respectively (Fig. 1). Notably, the *r*_1_ value of 1 is lower than our reported bishydrated MnCA (MnL^Me^),^1*a*^ and comparable to other monohydrated MnCAs reported,^16^ suggests that 1 is monohydrated. The increased *r*_1_ value of 2 is comparable to the *r*_1_ of the 1 : 2 instant complex 1 with Zn^2+^ (4.73 ± 0.50 mM^−1^ s^−1^) (Fig. S6, ESI†). This suggests that the DPA arms of 1 interact strongly with Zn^2+^ ions, helping transition from a mono to a bis-hydrated state and enhancing relaxivity.^17^ These promising results affirm the validity of the hypothesis and indicate that 1 exhibits high responsiveness to Zn^2+^, making it a potential contrast agent or probe for detecting and imaging Zn^2+^ in biological systems.^18^

**Fig. 1:**
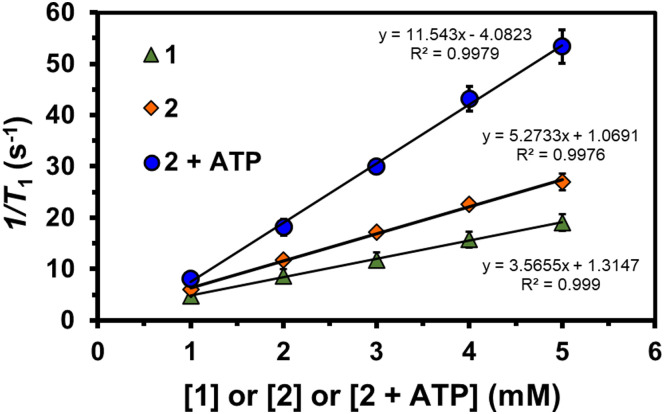
The plot of 1/*T*_1_*vs.* the concentration of 1, 2 and 2 + ATP (1–5 mM) in water or aqueous HEPES buffer solution (pH 7.3) at 298 K, 9.4 T.

Similarly, in the case of Zn-bound MnCA 2, adding one molar equivalent of ATP to the aqueous buffer solution (pH 7.3) led to a remarkable increase in relaxivity. The *r*_1_ value surged from 5.27 ± 0.23 to 11.54 ± 0.90 mM^−1^ s^−1^, indicating a strong interaction between ATP and 2. The substantial enhancement in relaxivity implies the involvement of second-sphere water ordering, potentially facilitated by potential hydrogen bonding interactions between solvated ATP and the outer-sphere water molecules. These results highlight the high responsiveness of 2 to ATP, suggesting its possible use as a CA or probe for detecting and imaging ATP in biological systems.^19^

The selectivity of 2 for ATP was further demonstrated by testing its response to other potentially interfering phosphates, such as inorganic phosphate (Pi), PPi, and adenosine mono- and diphosphates (AMP and ADP) (Fig. S6, ESI†). The *r*_1_ values for these phosphate adducts were found to be 3.69 ± 0.1, 1.11 ± 0.1, 4.38 ± 0.6, and 5.28 ± 0.2 mM^−1^ s^−1^, respectively, at 298 K, 9.4 T. Notably, the relaxivity induced by inorganic phosphates (Pi and PPi), AMP, and ADP was 4.8–10.4 times lower than that induced by ATP. When an equivalent of PPi was added to 2, its *r*_1_ value notably decreased, suggesting a strong binding between its Zn-DPA arms and PPi's phosphate groups. This intensely affected the Mn^2+^ hydration state, impeding water exchange, as seen with PPi-responsive GdCA.^15*a*^ Conversely, ADP caused minimal changes in relaxivity of 2, indicating no disturbance in its water exchange even when binding through both α and β phosphate groups in a 1 : 1 fashion.

Impressively, the proton relaxivity of 2 displayed a 2.2-fold increase upon adding an equivalent amount of ATP. This augmentation can be credited to binding β and terminal γ phosphate groups with the Zn^2+^-DPA units of 2 in a 1 : 1 ratio, leaving the α phosphate and adenosine groups free. This phenomenon is reminiscent of those reported in the literature for nucleotide-responsive GdCAs.^15*b*,*d*^ The α phosphate (p*K*_a_ = 1) and adenosine groups, oriented towards the solvent, will likely impact the swift water exchange between the Mn^2+^ centre and the surrounding solvent environment, significantly enhancing *r*_1_. In addition, the *r*_1_ value for free Mn^2+^ ions and ATP was also established as 8.8 ± 0.2 mM^−1^ s^−1^, 1.3 times lower than the relaxivity of 2. This rules out the leaching of Mn^2+^ ions upon the addition of ATP to 2 and provides further endorsement of the ATP-responsive behaviour of 2.

The Zn^2+^ and phosphate binding capabilities of 1 and 2 were evaluated using water proton relaxation enhancement (PRE) and absorption spectral titration methods (Fig. 2 and Fig. S5, ESI†). The phosphate-binding constants (*K*_b_) 2 obtained from absorption spectral titration profiles have followed this order: ATP > PPi > ADP > AMP > Pi (Table S1, ESI†). These findings confirm that 2 can selectively recognise ATP over other tested phosphate anions. The stronger *in vitro* ATP affinity of 2 might be emphasised *in vivo* due to cellular concentrations of ATP generally exceeding ADP.^20^ Further, *in vivo* studies are essential for validation.

**Fig. 2:**
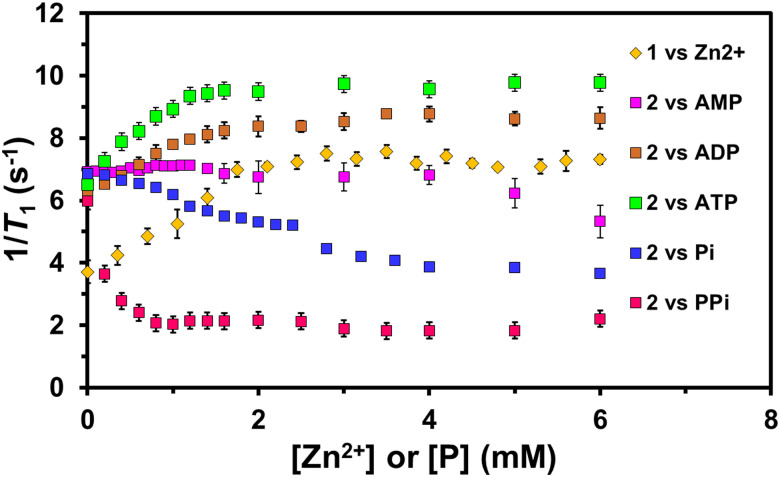
The plot of 1/*T*_1_*vs.* the function of increasing [Zn^2+^] and different phosphates [P] (where P = Pi, PPi, AMP, ADP, and ATP) (0–6 mM) for 1 (1 mM) and 2 (1 mM), respectively, in aqueous (50 mM) HEPES buffer solution at 298 K, 9.4 T.

To evaluate the kinetic and chelating stability of MnCAs (1 and 2), we performed transmetallation and ATP chelation experiments over 42 days (Fig. S7 and S8, ESI†). The 1 and 2 (1 mM) were incubated with 25 equivalent molar excess of Zn^2+^ and ATP in 50 mM HEPES (pH 7.3) at 298 K, 9.4 T. Initial water proton relaxation rates (*R*^obs^_1_ = 1/*T*_1_) for 1 and 2 were noted at 3.18 ± 0.1 and 5.22 ± 0.6 s^−1^, respectively. These rates rose to 5.38 ± 0.6 and 6.87 ± 0.11 s^−1^ upon adding excess Zn^2+^ and ATP. Interestingly, the *R*^obs^_1_ values of 1 and 2 with and without the presence of Zn^2+^ and ATP become almost constant over six weeks of the experiment, respectively. These results underscore the remarkable chelating stability of MnCAs 1 and 2, even in challenging conditions with excess competing agents.

A time-course absorption (UV-visible) spectral study was also conducted to demonstrate the chelating stability of 2 in an aqueous solution (50 mM HEPES buffer) at pH 7.3 over 23 days. 2 (50 μM) exhibited three distinct absorption bands around 217, 260, and 282 nm, with nearly constant initial *λ*_max_ values over time. To minimise measurement errors, the absorption maxima ratio *λ*_max_ values were plotted as a function of time (Fig. S9, ESI†). The calculated absorption intensity ratios (Abs_282nm_/Abs_260nm_) of 2 at pH 7.3 remained relatively constant over a week, indicating the compound's stability under physiological conditions.

To comprehend the potential of MnCAs as viable alternatives to GdCAs, we conducted a detailed examination of their pH-dependent relaxivity (*r*_1_), ensuring to include physiological pH (7.3) within the range from 3.2 to 10.5 (Fig. S10, ESI†) using 9.4 T at 298 K. The observed trends in the relaxivity values of the complexes at different pH levels provide exciting insights. At high pH, the deprotonation of water molecules occurs, leading to a drop in relaxivity. On the other hand, the possibility of free Mn ions arises at low pH by degradation. It is plausible that protonation and subsequent ordering of the second sphere of water molecules may influence the relaxivity enhancement behaviour.^18^

We found that complexes 1 and 2 significantly enhanced *r*_1_ values at lower pH levels compared to neutral conditions. Specifically, the *r*_1_ values between the pH values 3.2 and 7.3 for 1 were 5.85 ± 0.7, 4.01 ± 0.04, 3.79 ± 0.1, and 3.35 ± 0.2 mM^−1^ s^−1^, while those for 2 were 9.64 ± 0.4, 9.58 ± 0.62, 5.68 ± 0.2, and 5.16 ± 0.2 mM^−1^ s^−1^, respectively. This suggests that the spatial orientation of DPA/Zn arms may be vital in facilitating intramolecular hydrogen bonding between DPA nitrogens and the CONH-N moiety of 1. Alternatively, coordination of each CONH-N group with each DPA-Zn arm of 2 could also release free Mn^2+^, ultimately leading to increased relaxivity under acidic conditions. This contrasts with our previous report on the MnCA, MnL^Me^,^1*a*^ which showed only a slight decrease in relaxivity value at lower pH conditions due to the protonation of the carbohydrazone moiety (*K*_H_ > 13),^21^ stabilising the molecule through hydrogen bonding. However, at pH 8.5 and 10.5, the *r*_1_ values for 1 and 2 dropped to 2.21 ± 0.2, 1.98 ± 0.1 and 3.4 ± 0.1, 3.52 ± 0.2 mM^−1^ s^−1^, respectively. This gradual drop in *r*_1_ values suggests the formation of hydroxo complexes upon deprotonation that may partially or fully block water exchange and lead to a substantial decrease in water proton relaxivity.

To validate the MR contrasting potential of 1, 2 (with and without the presence of phosphates), Zn^2+^-DPA units-free MnCA (MnL^Me^)^1*a*^ and Magnevist® (clinically available GdCA, [Gd(DTPA)(H_2_O)]^2−^) by *T*_1_-weighted MR phantom images of the complexes at various concentrations (0.1–0.5 mM and 1–5 mM) were studied using 1.5 T clinical MRI system, respectively (Fig. 3 and Fig. S11, ESI†). The phantom images of the MnCAs 1, 2, and MnL^Me^ sample tubes showed higher signal intensity than the control experiments with water and Magnevist®. However, the responsive MnCA, such as 2, exhibited a significant increase in signal intensity upon selective binding with ATP over the phosphates mentioned above, aligning with the relaxivity trend at 9.4 T.

**Fig. 3:**
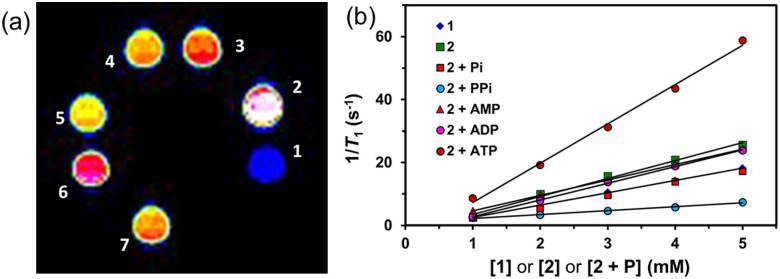
(a) *T*_1_-Weighted MRI phantom images (1.5 T, 298 K) of (1) water, (2) 2 + ATP, (3) 2 + PPi, (4) 2, (5) 1, (6) MnL^Me^ and (7) clinical standard Magnevist® (1 mM). (b) The plot of 1/*T*_1_ (s^−1^) *vs.* the concentration (mM) of 1, 2, and 2 + P (P = Pi, PPi, AMP, ADP, and ATP) in water at 1.5 T, 298 K.

The superior relaxivity of 2 + ATP at 1.5 T revealed a 2–5 fold and 10-fold enhancement (12.5 mM^−1^ s^−1^) than 2 (5.69 mM^−1^ s^−1^), 2 + AMP (4.9 mM^−1^ s^−1^), 2 + ADP (5.3 mM^−1^ s^−1^), 2 + Pi (3.76 mM^−1^ s^−1^), and 2 + PPi (1.23 mM^−1^ s^−1^), respectively. The enhanced relaxivity of 2 upon adding ATP is likely due to the formation of high molecular weight 2-ATP complex by coordination of β and γ-phosphates of ATP to the DPA-Zn^2+^ arms while leaving the α-phosphate uncoordinated in the space, leading to rapid water exchange and slowing down the tumbling rate. As a result, the 2-ATP's net charge becomes negative, facilitating enhanced relaxivity and a bright phantom image. Adding an equivalent of PPi to 2 results in a steady decline in relaxivity, possibly due to the 1 : 1 binding between the Zn-DPA arms, hindering water exchange without displacing Mn^2+^ coordinated water. However, adding a monophosphate such as Pi to 2 significantly increased the relaxivity due to removing hindrance through divergent 1 : 2 binding, as reported for GdCA.^15*a*^

In conclusion, we have synthesised and characterised two novel responsive MnCAs (1 and 2) incorporating Zn^2+^ and phosphate-recognising groups. Our investigations confirmed their chelation stability, structural properties, and excellent Zn^2+^ and ATP-responsive MRI contrast capabilities using a *T*_1_-weighted phantom imaging strategy. Our results demonstrate that responsive MnCAs 1 and 2 have superior water proton relaxivity (*r*_1_) values of 3.35 and 5.27 mM^−1^ s^−1^, respectively, compared to clinically available GdCA such as [Gd(DTPA)(H_2_O)]^2−^ (Magnevist®) and our previously reported MnCA (MnL^Me^).^1*a*^ Moreover, upon binding with Zn^2+^ and ATP over other phosphates, the *r*_1_ values of 1 and 2 were significantly enhanced to 4.73 and 12.52 mM^−1^ s^−1^ at 1.5 T, respectively. Overall, the results presented in this study provide important insights into the development of highly efficient MRI contrast agents for diagnostic applications.

SA gratefully acknowledges the Departments of Chemistry and Biomedical Sciences, the University of Hull, for providing laboratory space and instruments access to carry out this work. The work has also been partially supported by the RSC Research Fund grant (RF19-7464). GJS would like to thank the MRC (MR/T002573/1) and the EPSRC (EP/V027549/1 and EP/T026367/1) for funding this work.

## Conflicts of interest

There are no conflicts to declare.

## Supplementary Material

CC-059-D3CC03430E-s001
