# Supplementary material for: ATP-responsive Mn(ii)-based T1 contrast agent for MRI
Source: Chem Commun (Camb). 2023 Sep 19;59(91):13623–6. doi: 10.1039/d3cc03430e (PMC10644988; doi:10.1039/d3cc03430e)
Supplement: CC-059-D3CC03430E-s001 [file CC-059-D3CC03430E-s001.pdf]

Electronic Supplementary Material (ESI) for Chemical Communications.
This journal is © The Royal Society of Chemistry 2023

ATP-responsive Mn(II)-based T_1 contrast agent for MRI†

Sellamuthu Anbu,*[,a,b] Lawerence Kenning[c] and Graeme J. Stasiuk*[,d]

[a]Departments of Chemistry and Biomedical Sciences, University of Hull, Cottingham Road, Hull, HU6 7RX, UK.

E-mail: bioinorg_anbu@yahoo.com

[b]School of Chemistry, University of East Anglia, Norwich Research Park, Norwich, NR4 7TJ, UK.

[c]MRI centre, Hull Royal Infirmary Hospital NHS Trust, Anlaby Road, Hull, HU3 2JZ, UK.

[d]Department of Imaging Chemistry and Biology, School of Biomedical Engineering and Imaging Sciences, King's College London, Fourth Floor Lambeth Wing, St Thomas' Hospital, London, SE1 7EH, UK.

E-mail: graeme.stasiuk@kcl.ac.uk

Materials and Measurements

Experimental Procedures

Figs. S1–S4 ^{1}H, ^{13}C NMR and Electrospray-Mass spectral profiles of compounds **DPACOHz**, **MnL^{DPA}** (**1**) and **MnL^{DPA-Zn}** (**2**).

Fig. S5. Absorption spectral titration profiles of Mn^{2+} complexes **1** with Zn^{2+} and **2** with Pi, PPi, AMP, ADP, and ATP.

Fig. S6. T_1 relaxation rate ($1/T_1$, s^{-1}) as a function of the concentration of **1** + Zn^{2+}, **2**, **2** + Pi, **2** + PPi, **2** + AMP, **2** + ADP and Mn^{2+} + ATP (1-5 mM).

Fig. S7. The ATP-chelation of **1** mM of **2** by 25 mM molar excess equivalent of ATP was monitored by relaxivity change as a function of time.

Fig. S8. Transmetallation of 1 mM responsive MnCA (**1**) by 25 mM Zn^{2+} and kinetic stability of **2** monitored by the change in $1/T_1$ (s^{-1}) over six weeks in water at 298 K, 9.4 T.

Fig. S9. The absorption spectral method monitored the chelating stability of **2**.

Fig. S10. pH-dependent relaxivity of **1** and **2**.

Fig. S11. T_1-weighted MRI phantom images (1.5 T, 298 K) as a function of the concentration of MnCAs and different phosphates.

Table S1. Selected best-fit parameters obtained from the analysis of the Relaxometry and absorption spectral titration data of **1** and **2** and their Zn^{2+} and sodium salts of Pi, PPi, AMP, ADP, and ATP complexes.

References

Materials and Measurements

The precursor compound 2-(bis(pyridin-2-ylmethyl)amino)acetohydrazide **(DPACOHz)** was synthesised by employing a slightly modified literature-reported procedure.[1] All materials and reagents for synthesising Mn^{2+} (**1** and **2**) complexes were purchased from Sigma-Aldrich (Dorset, UK) and used without further purification. Solvents were purchased from VWR (Leicestershire, UK). IR spectra were recorded on a Perkin Elmer Rx FTIR x2 with diamond ATR and DRIFT attachment. ^{1}H-NMR and T_1 measurements were carried out on a JEOL JNM-LA400 Spectrometer (400 MHz). Electronic absorption spectral titration experiments were done using a ThermoFisher Scientific Evolution 300 UV-Vis spectrophotometer. Electrospray ionisation mass spectrometry (ESI-MS) spectra of the compounds were recorded on Advion MS SOP electrospray ionisation (ESI) spectrometer. The pH measurements were done using a Jenway 3520 digital pH meter with a Mettler-Toledo 51343160 glass electrode.

Synthesis of 2-(bis(pyridin-2-ylmethyl)amino)acetohydrazide (DPACOHz). Glycine ethyl ester hydrochloride (1.39 g, 9.96 mmol) was mixed with 2-chloromethyl pyridine hydrochloride (3.27 g, 19.9 mmol) and K_2CO_3 (2.88 g, 20.9 mmol) in dry acetonitrile (60 ml) and refluxed overnight. The resulting mixture was hot filtered and removed the solvent by vacuum. The dark brown oily residue was dissolved in ethyl acetate (10 mL) and washed with water (2 × 50 mL), and the organic phase was dried in Na_2SO_4. A thick brown oil was isolated by removing the solvent by vacuum used without further purification and refluxed with hydrazine hydrate (10 ml) for 48 h in ethanol (60 mL). The solvent was removed by vacuum, and the resulting oily-like product was dissolved in chloroform (40 mL), washed with copious amounts of water to remove the excessive hydrazine hydrate and dried in anhydrous Na_2SO_4. A dark yellow oily product is isolated by removing the solvent by vacuum. Yield: 1.75 g (65%). ^{1}H NMR ($CDCl_3$, 400 MHz) δ (ppm): 10.03 (s, 1H), 8.51 (dd, 2H, J = 2.1, 1.8 Hz, H-Pyridine), 7.54 (ddd, 2H, J = 1.8, 1.7, 1.8 Hz, H-Pyridine), 7.19 (d, 2H, J = 7.8 Hz, H-Pyridine), 7.10 (t, 2H, 6.0, 6.2 Hz, H-Pyridine), 3.76

(s, 4H, H-methylene), 3.31 (s, 2H, H-methylene). ^{13}C NMR ($CDCl_3$, 100 MHz) δ (ppm): 57.36, 60.51, 122.57, 123.36, 136.75, 149.46, 158.07, and 171.26. ESI-MS (CH_3OH): Calcd. for $C_{14}H_{18}N_5O$, 272.15 found 272.17.

Synthesis of Mn^{2+} complex MnL^{DPA} (1). The DPA-functionalised Mn^{2+} complex **1** was synthesised by following the procedure we reported[2] for **MnL^{Me}** by refluxing 2,6-diacetylpyridine (DiAcPy) (0.163 g, 1.0 mmol) and $MnCl_2$ (0.121 g, 0.96 mmol) with 2-(bis(pyridin-2-ylmethyl)amino)acetohydrazide (**DPACOHz**) (0.543 g, 2.0 mmol) instead of acetohydrazide in methanol. The resultant mixture was refluxed overnight and hot-filtered. After cooling the pale orange solution to room temperature, the solvent was removed by vacuum. The resulting orange oil was washed with small amounts of cold methanol and dried. The obtained water-soluble oily product was recrystallised from MeOH. Isolated yield: 0.59 g (85%). IR (KBr, cm^{-1}): ν 3483s (N−H), 1676s (C=O), 1635s (C=N), 1451vs (N−O), 1252s (N−O), 1020s (−N−N). HRMS: Calcd. for $C_{37}H_{38}MnN_{11}O_2$ 723.2579 found 723.2599 and calcd. for $C_{37}H_{38}MnN_{11}NaO_2$ 745.2599 found 745.2404.

Synthesis of Mn^{2+} complex $MnL^{DPA\text{-}Zn}$ (2). An ethanolic solution (10 mL) of $ZnCl_2$ (0.11 g, 0.8 mmol) was added dropwise to the complex **1** (0.3 g, 0.4 mmol) in ethanol (40 mL) under constant stirring at 50 °C. After 3 h of stirring, a creamy white precipitate was isolated by filtration, washed with cold ethanol, and dried under a vacuum. The solid is suspended in acetone/water, and the solvent was evaporated by vacuum. A creamy white foamy solid product was isolated and recrystallised from water and acetonitrile (2:1, v/v) mixture. Isolated yield: 0.34 g (82%). IR (KBr, cm^{-1}): ν 3450s (N−H), 1665s (C=O), 1642s (C=N), 1452vs (N−O), 1240s (N−O), 1025s (−N−N). HRMS: Calcd. for $C_{37}H_{37}Cl_3MnN_{11}O_2Zn_2$, $[MnL^{DPA\text{-}Zn}\text{-}Cl^-]^+$, 959.0286 found 959.0108 and $C_{37}H_{37}Cl_2MnN_{11}O_2Zn_2$, $[MnL^{DPA\text{-}Zn}\text{-}2Cl^-]^{2+}$, 462.1932 found 462.0211.

Relaxometry studies. The longitudinal relaxation times (T_1) of the **MnL^{DPA} (1)** and **$MnL^{DPA\text{-}Zn}$ (2)** were recorded on a JEOL JNM-LA 400 Spectrometer (400 MHz). The T_1 values were obtained by the inversion-recovery method (180° − τ − 90°). The relaxivity of the complexes was

ascertained by different concentrations of **1** and **2** (1.0 to 5.0 mM) at pH = 7.3 (50 mM HEPES buffer, 0.15 M NaCl, 298 ± 0.2 K). The water proton relaxivity r_1 values of **1** and **2** were calculated from the slope of the plot of $1/T_1$ versus [**MnL$^{DPA/Zn}$**]. Stock solutions of **1** and **2** (10 mM) and sodium salts of phosphate (Pi), pyrophosphate (PPi), adenosine mono, di and triphosphate (10 mM) were prepared in MilliQ water.

MR Imaging. The T_1-weighted phantom MR images of the complexes **MnL^{DPA}** (**1**) and **MnL^{DPA-Zn}** (**2**) were performed at concentrations between 1 and 5 mM in 50 mM HEPES buffer, pH 7.3. An additional set of solutions was made with **MnL^{DPA-Zn}** (2) and phosphate biomolecules (Pi, PPi, AMP, ADP, ATP) at a concentration of 0.6 mM in the same buffer. These were prepared in Eppendorf tubes and placed in a tray, each with a volume of 1.0 mL. Phantom imaging was executed on a 1.5 T GE Signa clinical MR scanner. 3D T1-FLASH sequences were used to acquire T_1-weighted MR images of samples **1** and **2** (both with and without Zn^{2+} and the phosphates). The imaging parameters were TE = 2.64 ms, TR = 8.78 ms, flip angle 30°, FOV 60x40x21.21 mm^3, image size 272x136x96, slice thickness 21.12 mm, resolution 0.220x0.294x0.220 mm^3, and an average total acquisition time of 1 min 54 s. For the comparative study with Mn(II) chelates like **MnL^{Me}** and the clinically applied GdCA (Magnevist^{TM}), the flip angle was adjusted to 10° in T_1-weighted imaging. T_1 maps were obtained using a 2D RAREVTR sequence, TE = 6.705 ms, FOV = 50x50 mm^2, image size = 128x128, resolution = 0.390x0.390 mm^2, with a total scan time of 12 min 52 s. The sequence was performed with 15 TR values from 50 to 3000 ms.

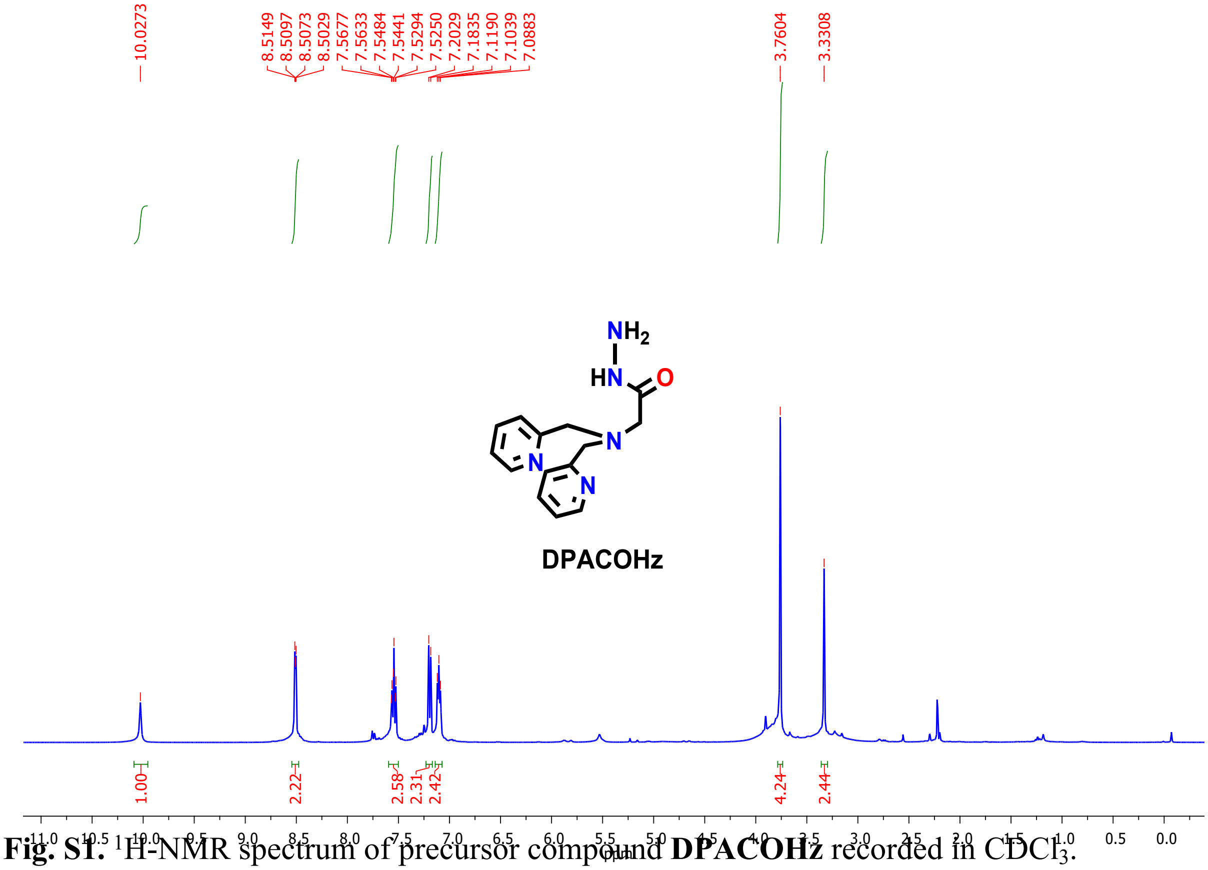

Fig. S1. ^{1}H-NMR spectrum of precursor compound **DPACOHz** recorded in $CDCl_3$.

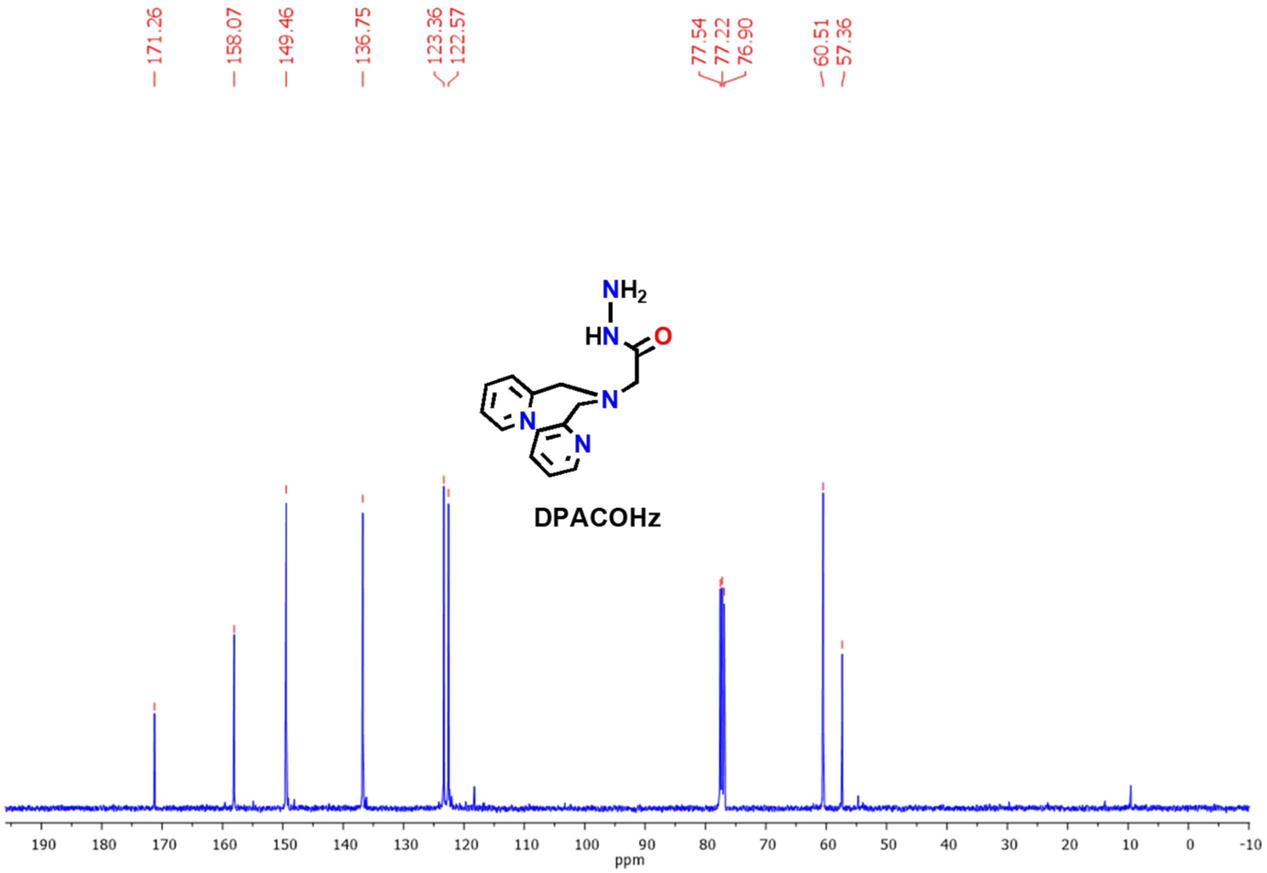

Fig. S2. ^{13}C-NMR spectrum of precursor compound **DPACOHz** recorded in $CDCl_3$.

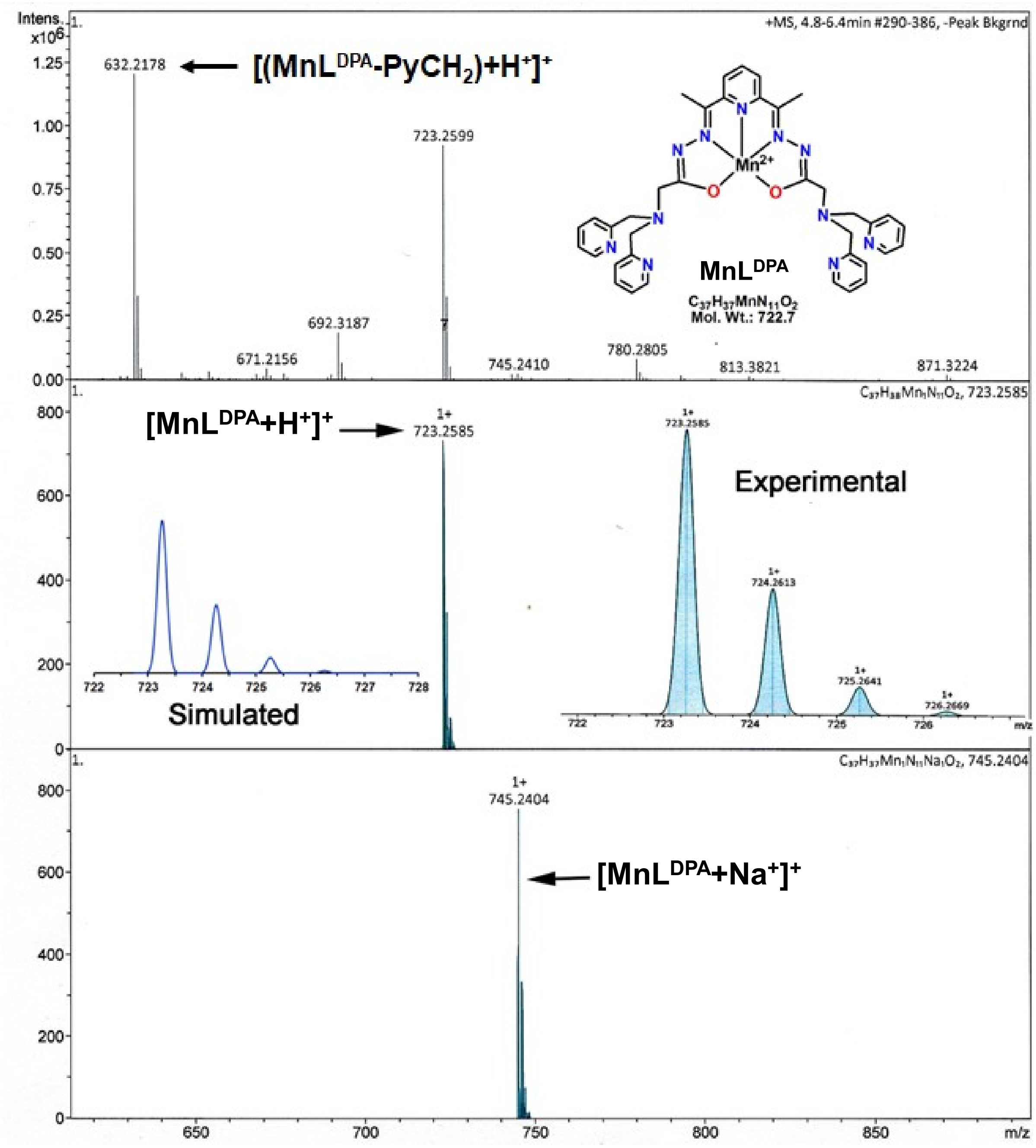

Fig. S3. HRMS spectrum of **MnL^{DPA}** (**1**) recorded in CH_3OH. The peaks (*m/z*) at 632.2178, 723.2585, and 745.2404 correspond to [MnL^{DPA}-PyCH$_2$)+H$^+$]$^+$ (Py-CH$_2$: pyridyl methyl, C_4H_4N-CH_2), [MnL^{DPA}+ H$^+$]$^+$, and [MnL^{DPA}+ Na$^+$]$^+$ species, respectively.

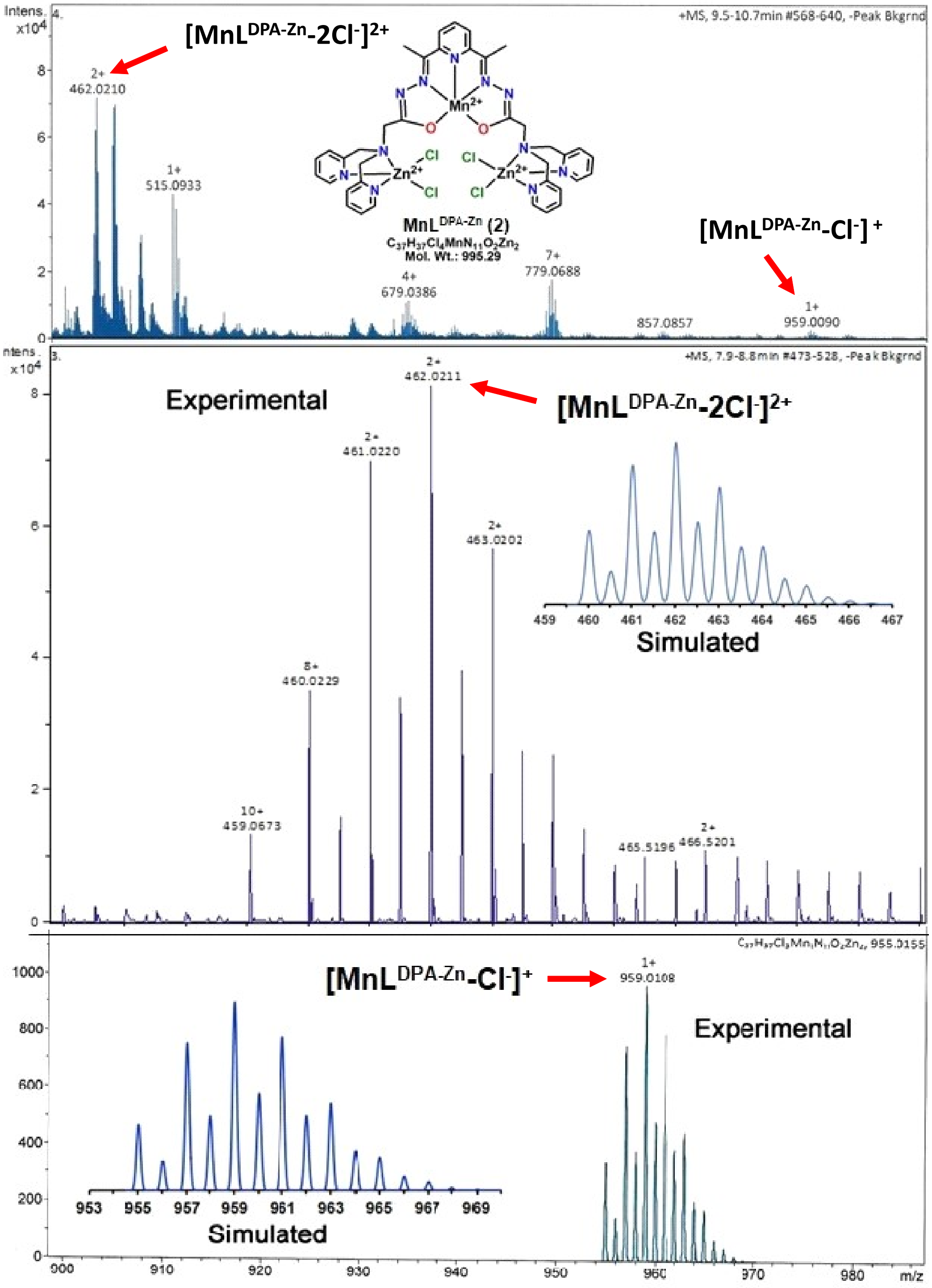

Fig. S4. HRMS spectrum of **MnL$^{DPA\text{-}Zn}$** (**2**) recorded in CH_3OH. The peaks (*m/z*) at 462.0211 and 959.0108 correspond to [MnL$^{DPA\text{-}Zn}$-2Cl$^-$]$^{2+}$ and [MnL$^{DPA\text{-}Zn}$-Cl$^-$]$^+$ species, respectively.

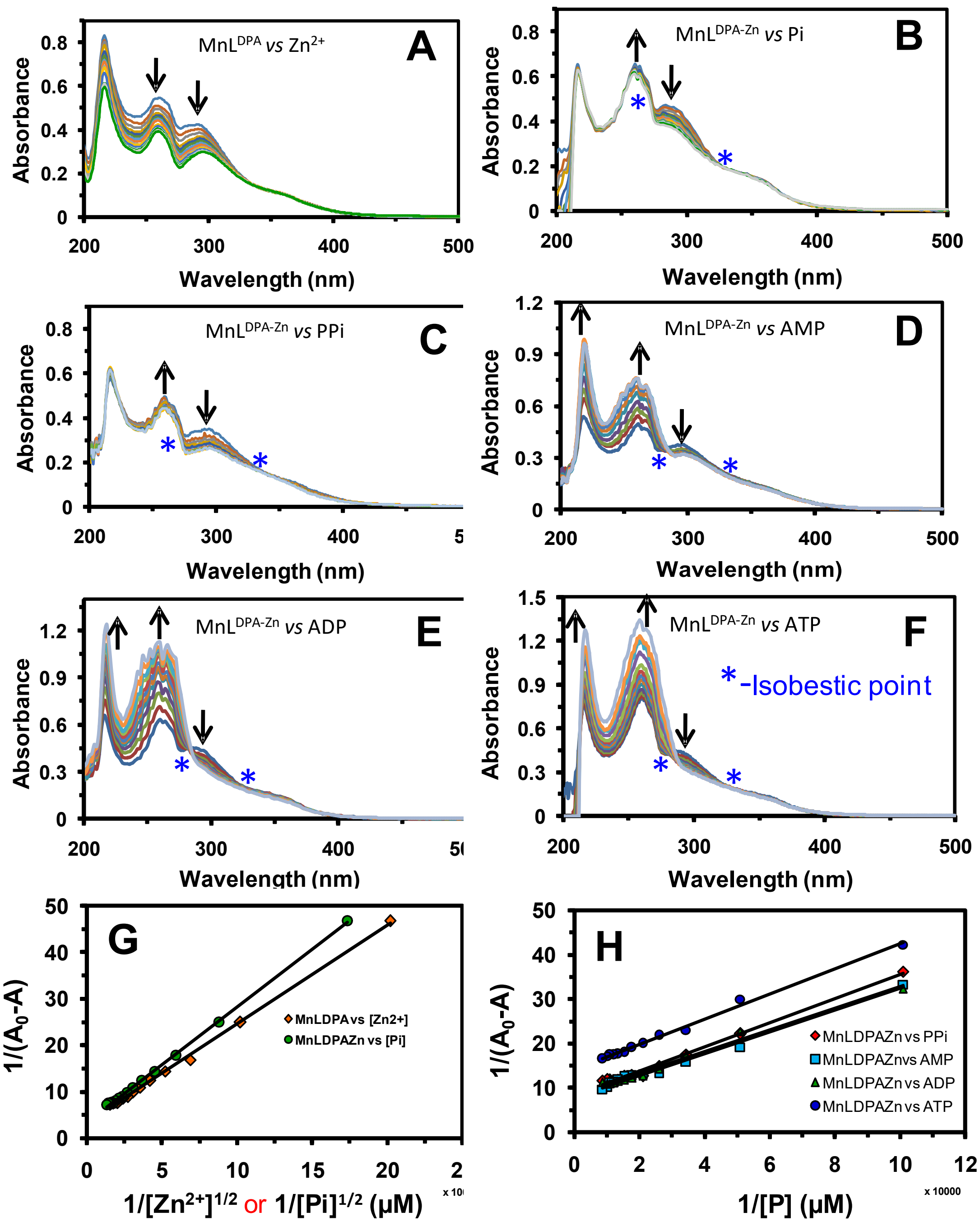

Fig. S5. (A-F) Absorption spectral titration profiles of **MnL^{DPA}** (**1**) and **MnL^{DPA-Zn}** (**2**) (50 μM) with Zn^{2+} (0−150 μM) and sodium salts of Pi (0−150 μM), PPi (0−120 μM), AMP (0−150 μM), ADP (0−150 μM), and ATP (0−120 μM), respectively, in 50 mM HEPES buffer, pH 7.3. (G and H) Benesi-Hildebrand plots of $1/(A_0\text{-}A)]$ as function of $1/[Zn^{2+}]^{1/2}$ or $1/[Pi]^{1/2}$, and $1/(A_0\text{-}A)]$ vs 1/[P], respectively, where P = PPi, AMP, ADP and ATP.

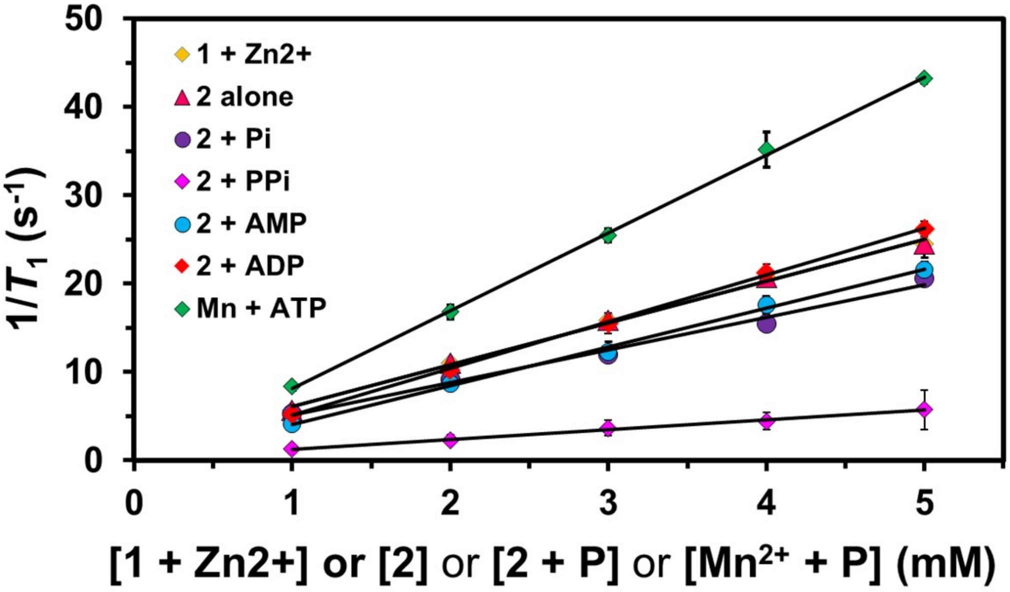

Fig. S6. T_1 relaxation rate ($1/T_1$, s^{-1}) as a function of the concentration of **1** + Zn^{2+}, **2**, **2** + Pi, **2** + PPi, **2** + AMP, **2** + ADP and Mn^{2+} + ATP (1-5 mM) in aqueous (50 mM HEPES) buffer solution (pH 7.3) at 298 K, 9.4 T.

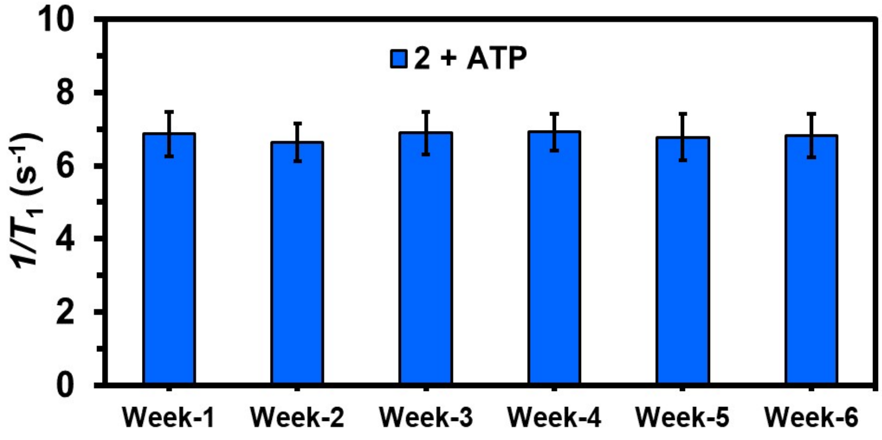

Fig. S7. The ATP-chelation of 1 mM of **MnL$^{\mathbf{DPA\text{-}Zn}}$** (**2**) by 25 mM molar excess equivalent of ATP was monitored by relaxation rate ($1/T_1$, s^{-1}) change over six weeks in an aqueous solution (50 mM HEPES buffer) at pH 7.3, 298 K, 9.4 T.

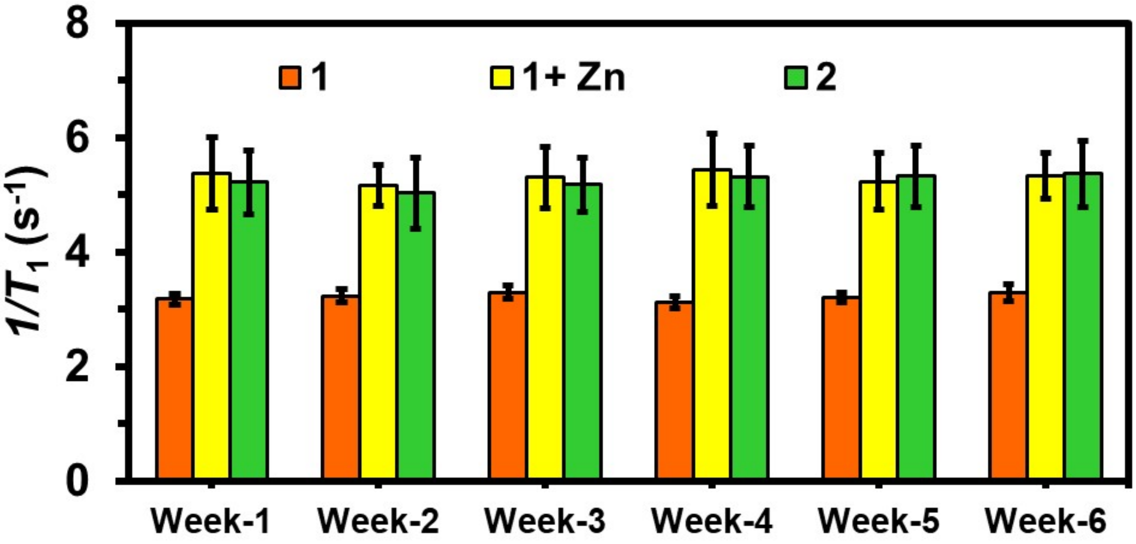

Fig. S8. Transmetallation of 1 mM responsive MnCA (**1**) by 25 mM Zn^{2+} and kinetic stability of **2** monitored by the change in $1/T_1$ (s^{-1}) over six weeks in water at 298 K, 9.4 T.

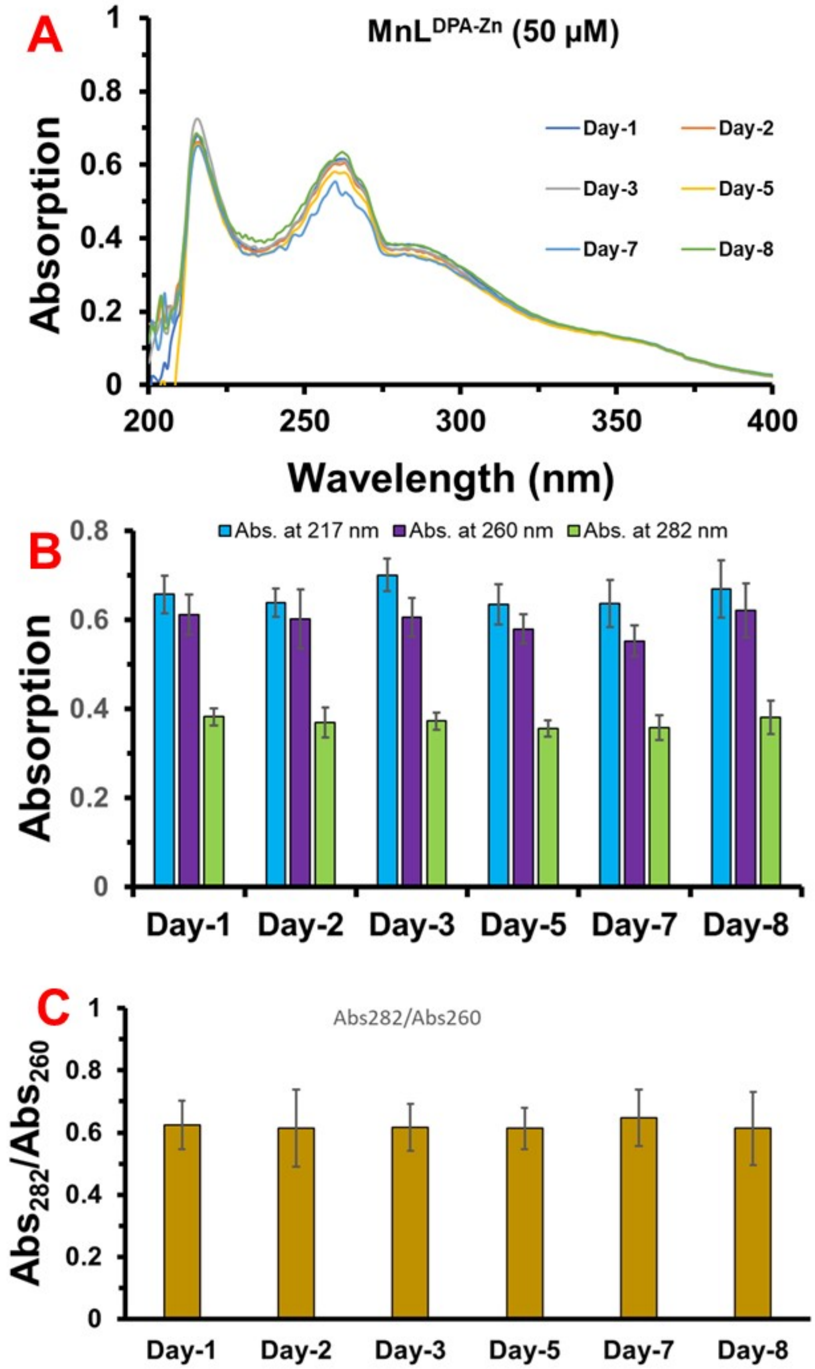

Fig. S9. The chelating stability of **2** (**MnL^{DPA-Zn}**) was monitored by a change in the absorption spectral intensity over time in an aqueous solution (50 mM HEPES buffer) at pH 7.3, 298 K. Absorption spectra of **2** (50 µM) obtained over 8 days (A), Change in the absorption intensities at 217, 260, and 282 nm (B), and the ratio of Abs_{282}/Abs_{260} as the function of time (C).

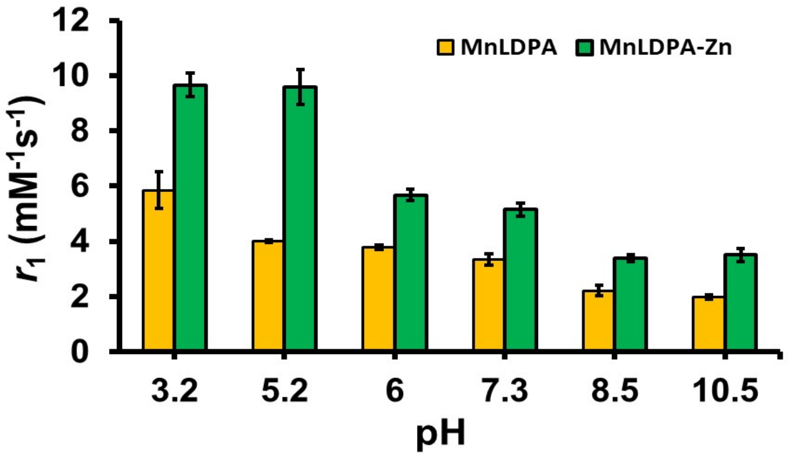

Fig. S10. Change in relaxivity of **MnL^{DPA}** (**1**) and **MnL$^{DPA\text{-}Zn}$** (**2**) (1 mM) in 50 mM aqueous (phosphate/phosphoric acid (pH 3.2), MES (pH 5.2 and 6.0), HEPES (pH 7.3), Potassium Phosphate/KOH (pH 8.5), NH_4Cl/NH_3 (pH 10.5) buffer solutions, at 298 K, 9.4 T.

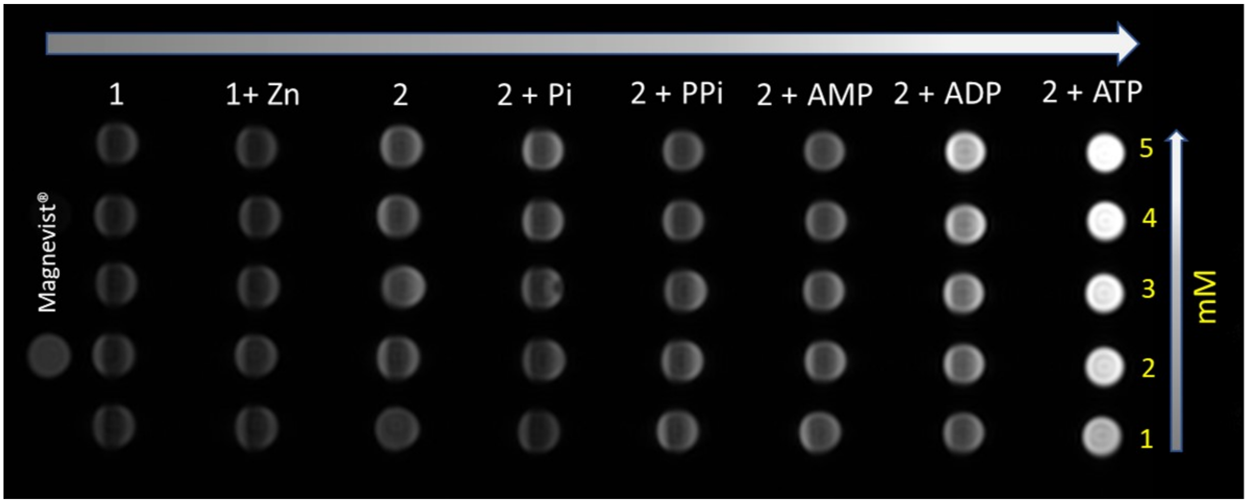

Fig. S11. T_1-weighted MRI phantom images (1.5 T, 298 K) as a function of the concentration of Magnevist® (2 mM), **1**-alone, **1** + Zn^{2+}, **2**-alone, **2** + Pi, **2** + PPi, **2** + AMP, **2** + ADP, and **2** + ATP (1 to 5 mM) in an aqueous (HEPES) buffer solution, pH 7.3, 298 K, 1.5 T.

Table S1. Selected best-fit parameters obtained from the analysis of the Relaxometry and absorption spectral titration data of **1** and **2** and their Zn^{2+} and Pi, PPi, AMP, ADP, and ATP complexes in an aqueous (HEPES) buffer solution, pH 7.3.

**MnCA**	r_1 **(mM^{-1}.s^{-1})^a**	r_1 **(mM^{-1}.s^{-1})^b**	**Binding Constant (K_a)^c**
MnL^{DPA} (**1**)	3.35 ± 0.20	3.72	-
MnL^{DPA} (**1**) + Zn	4.73 ± 0.50	-	-
$MnCl_2$ + ATP	8.80 ± 0.20	-	-
MnL^{DPA-Zn} (**2**)	5.27 ± 0.09	5.69	3.41×10^8 M^{-2}
MnL^{DPA-Zn} (**2**) + Pi	3.691 ± 0.1	3.76	2.50×10^8 M^{-2}
MnL^{DPA-Zn} (**2**) + PPi	1.112±0.1	1.23	4.15×10^4 M^{-1}
MnL^{DPA-Zn} (**2**) + AMP	4.384±0.6	5.69	3.26×10^4 M^{-1}
MnL^{DPA-Zn} (**2**) + ADP	5.278±0.2	5.31	3.42×10^4 M^{-1}
MnL^{DPA-Zn} (**2**) + ATP	11.54 ± 0.90	12.5	7.41×10^4 M^{-1}

The r_1 parameters obtained at 9.4 T^a and 1.5 T^b, 25 °C. ^{c}Values obtained from absorption spectral titration profiles.

References:

(1) T. Ganguly, B. B. Kasten, D.-K. Bucar, L. R. MacGillivray, C. E. Berkman, and P. D. Benny, The hydrazide/hydrazone click reaction as a biomolecule labelling strategy for $M(CO)_3$ (M = Re, ^{99m}Tc) radiopharmaceuticals. *Chem. Commun*. **2011**, *47*, 12846–12848.

(2) S. Anbu, S. H. L. Hoffmann, F. Carniato, L. Kenning, T. W. Price, T. J. Prior, M. Botta, A. F. Martins and G. J. Stasiuk, *Angew. Chem. Int. Ed*., **2021**, 60, 10736–10744.

(3) Blessing, R. H. An empirical correction for absorption anisotropy, *Acta Crystallogr., Sect. A: Found. Crystallogr*. **1995**, *51*, 33–38.
